# Machine learning-based predictive model for acute pancreatitis-associated lung injury: a retrospective analysis

**DOI:** 10.3389/fmed.2025.1638097

**Published:** 2025-08-12

**Authors:** Zhaohui Du, Qiaoling Ying, Yisen Yang, Huicong Ma, Hongchang Zhao, Jie Yang, Zhenjie Wang, Chuanming Zheng, Shurui Wang, Qiang Tang

**Affiliations:** ^1^Department of Emergency Surgery, The First Affiliated Hospital of Bengbu Medical University, Bengbu, Anhui, China; ^2^Department of Radiation Oncology, The First Affiliated Hospital of Bengbu Medical University, Bengbu, Anhui, China; ^3^Department of Epidemiology and Biostatistics, Institute of Basic Medical Sciences Chinese Academy of Medical Sciences, School of Basic Medicine Peking Union Medical College, Beijing, China; ^4^The Second Affiliated Hospital of Zhejiang University School Medicine, Hangzhou, China

**Keywords:** prediction model, machine learning, SHAP, acute pancreatitis (AP), lung injury

## Abstract

**Background:**

Acute Pancreatitis-Associated Lung Injury (APALI) is one of the most severe and life-threatening systemic complications in acute pancreatitis patients, with high rates of morbidity and mortality. This study aims to develop a prediction model for the diagnosis of APALI based on machine learning algorithms.

**Methods:**

This study included data from the First Affiliated Hospital of Bengbu Medical College (July 2012 to June 2022), which were randomly categorized into the training and testing set. And data from the Second Affiliated Hospital of Zhejiang University (January 2018 to April 2023) served as the external validation set. LASSO regression was applied to eliminate irrelevant or highly collinear independent variables. Six machine learning models were constructed, with evaluation metrics including Area Under Curve (AUC), accuracy, sensitivity, specificity, F1 score, and recall. The impact of model features was analyzed using SHapley Additive exPlanations (SHAP).

**Results:**

A total of 1,975 patients with acute pancreatitis were randomly assigned to a training set (1,480 patients) and a testing set (495 patients). In the training set, 480 cases (32.43%) were diagnosed with APALI. The eXtreme Gradient Boosting (XGBoost) and Random Forest (RF) models demonstrated the best predictive performance, achieving the highest AUC (0.92 and 0.914, respectively), along with higher accuracy, F1 score, and recall in the testing set. Six particularly influential factors were identified and ranked as follows: CRP, BMI, neutrophil, calcium, lactate, and neutrophil-to-albumin ratio (NAR). The global interpretability of the XGBoost and RF models, along with these six features, is shown in the SHAP summary plot. These two models were selected as the optimal models for the development of an online calculator for clinical applications and risk stratification.

**Conclusion:**

We developed and internally validated a machine learning model to predict APALI, showing strong performance in our study population. To support further research and clinical use, we created an open-access web-based risk calculator. Prospective multicenter validation is needed to confirm generalizability. If successful, the tool may support early risk identification and guide interventions to prevent APALI.

## Introduction

Acute pancreatitis is characterized by abdominal pain, distension, nausea, vomiting, and systemic complications. The incidence of AP has been steadily increasing, with approximately 20% of patients progressing to severe acute pancreatitis (SAP) (1, 2). SAP can lead to a range of complications, among which acute lung injury is particularly severe (3), highlighting the need for early prediction of such outcomes. A multicenter retrospective study demonstrated that 92% of SAP may develop acute respiratory distress syndrome (ARDS), with a mortality rate of 37% (4), which underscores the clinical importance of identifying high-risk patients at an early stage. If not properly managed, ARDS can progress to multiple organ failure (MOF), which poses a significant threat to the patient’s life (5). Despite the availability of various treatments for acute pancreatitis-associated acute lung injury (APALI), the mortality and morbidity rates remain high (6). Timely identification and intervention could mitigate or even alleviate acute lung injury, reducing both patient suffering and the economic burden (7, 8). Therefore, developing a clinical prediction model that can accurately identify lung injury at an early stage in AP is essential for improving patient outcomes. Given the multifaceted nature of acute lung injury, characterized by diverse clinical and biological features, no single clinical indicator can adequately represent the disease status (9). Recently, few studies have established an prediction model to identify APALI in patients with AP, for instance, Samanta et al. (10) proposed IL-6 and IL-8 as potential biomarkers for lung injury in AP, while Jia et al. (11) developed a nomogram-based tool using routine clinical data—however, both studies were limited by small sample sizes and lacked external validation. These limitations provided a strong rationale for the development of a robust, interpretable, and externally validated machine learning model as we present in this study.

In recent years, machine learning (ML) has been increasingly applied in critical care, offering notable advantages over conventional statistical methods. Evidence indicates that ML contributes significantly to the early diagnosis, severity assessment, and personalized treatment of AP (12–14). Algorithms such as logistic regression, random forests, and support vector machines each exhibit unique strengths in processing medical data (15, 16). Ong et al. demonstrated the utility of ML by developing an XGBoost-based model that outperformed traditional methods in predicting adverse outcomes—including readmission, mortality, and prolonged hospitalization—among patients requiring mechanical ventilation for more than 4 hours. The model achieved an AUC of 0.693 compared to 0.667 for conventional approaches (*p* = 0.03) and showed a 6.8% improvement in sensitivity at 95% specificity. Their study also highlighted important predictors, such as the Glasgow Coma Scale and duration of mechanical ventilation, while emphasizing the critical role of external validation and model interpretability in AI-driven applications within critical care (17). Although current ML models have limited accuracy in predicting APALI, ongoing technological advancements and the growing availability of electronic medical records are expected to enhance the precision and timeliness of clinical decision support—particularly for identifying high-risk patients and supporting individualized risk assessments.

This study developed a clinical prediction model for APALI using machine learning algorithms and validated its performance on external datasets. By leveraging extensive clinical data, the model identified critical indicators associated with APALI risk, providing early warnings for physicians. This research may accelerate the early prediction of lung injury in AP and supports medical teams in implementing targeted interventions, ultimately improving recovery rates and quality of life.

## Methods

### Study population

This study included AP cases admitted to the Emergency Surgery Department of the First Affiliated Hospital of Bengbu Medical University from July 2012 to June 2022, which were used for both the training and validation cohorts. Additionally, cases from the Second Affiliated Hospital of Zhejiang University, admitted between January 2018 to April 2023, served as the external validation cohort. All patient data were de-identified prior to analysis. The study was approved by the ethics committees of both participating hospitals (2020KY073), and informed consent was obtained from all enrolled patients or their legally authorized representatives in accordance with the Declaration of Helsinki.

Inclusion Criteria: Diagnosis of AP was based on the consensus of the International Association of Pancreatology (IAP), requiring at least three of the following conditions: typical clinical manifestations (e.g., abdominal pain); elevated serum amylase and/or lipase levels (generally exceeding three times the normal value), and imaging findings consistent with pancreatitis. Exclusion Criteria: Traumatic pancreatitis, acute exacerbation of chronic obstructive pulmonary disease (AECOPD), ARDS due to causes other than pancreatitis, and age under 14 years.

### Definition

According to the guidelines of the American Thoracic Society (ATS) and the European Respiratory Society (ERS), acute lung injury is defined as follows (18–21): (1) rapid onset, with acute respiratory distress typically occurring within hours; (2) hypoxemia is defined as a PaO2/FiO2 ratio of 200–300 mmHg; (3) Pulmonary infiltrates on chest X-rays or CT scans, excluding cardiogenic causes; and (4) The absence of cardiogenic pulmonary edema confirms that the injury is not due to heart failure or fluid overload. Diagnosis of acute lung injury was confirmed independently by two board-certified radiologists who were blinded to patient clinical outcomes. In cases of disagreement, a third senior radiologist adjudicated the final classification by consensus. Standardized diagnostic criteria were uniformly applied across all cases.

### Data collection

Clinical data were collected from patients with AP, including vital signs, demographic information, CT grade, and laboratory makers such as calcium (Ca^2+^), blood glucose (BG), lactate (Lac), serum lipase (LPS), serum amylase (AMY), urinary amylase (UAMY), triglycerides (TG), total cholesterol (TC), procalcitonin (PCT), heparin-binding protein (HBP), C-reactive protein (CRP), albumin, globulin, and the neutrophil-to-albumin ratio (NAR), Data on the presence of mechanical ventilation, oxygen partial pressure, concentration of inspired oxygen, and pleural effusion were also collected.

All laboratory variables were standardized to be collected within 24 h of hospital admission to ensure temporal consistency across patients. Elective admissions were excluded from the analysis to avoid confounding due to different baseline risk profiles and disease progression patterns. CT imaging was independently reviewed by two board-certified radiologists with 5–10 years of experience in abdominal imaging. Severe AP was defined as a CT Severity Index (CTSI) score ≥5, in accordance with the Revised Atlanta Classification. Discrepancies in CT grading were resolved by consensus with a senior radiologist with over 15 years of clinical experience. All CT evaluations and clinical/laboratory data were obtained within the first 24 h of hospital admission. Despite the class imbalance, no oversampling or class weighting was applied during analysis.

### Model construction and evaluation

To deploy the clinical predictive model, we first applied 10-fold cross-validated LASSO regression for preliminary feature selection. This step aimed to eliminate variables with low contributions and address multicollinearity among highly correlated predictors. The number of selected variables was determined using the *λ* that minimized the cross-validation error. These selected features were then used as inputs for subsequent machine learning models.

We constructed six machine learning models, including Logistic Regression (LR), Random Forest (RF), Extreme Gradient Boosting (XGBoost), Support Vector Machine (SVM), K-Nearest Neighbors (KNN), and Neural Network (NNET). During the data preprocessing stage, missing values were imputed with either the median or mode, depending on the variable type, and categorical variables were encoded using one-hot encoding. The dataset was split into training and test sets in a 3:1 ratio. Hyperparameter optimization was conducted using grid search with 25 bootstrapped resamples of the training set to ensure robust tuning.

Model performance was assessed using multiple metrics, including the area under the receiver operating characteristic curve (AUC), accuracy, sensitivity, specificity, F1 score, and recall. To further ensure reliability, external validation data were used, and AUC, accuracy, sensitivity, specificity, F1 score, and recall were recalculated.

### Model interpretation

To clarify the contribution of each feature to the final model, we used Shapley Additive Explanations (SHAP) to interpret and visualize the impact of individual variables. We assessed feature importance by calculating the mean absolute SHAP values. Additionally, we plotted SHAP values for each feature across all samples to better understand the overall patterns and the influence of features on the dataset. Three SHAP examples were provided to illustrate these concepts.

### Statistical analysis

Binary variables were summarized as counts and proportions, and comparisons were performed using chi-square tests or Fisher’s exact tests, as appropriate. Continuous variables with a normal distribution were compared using independent t-tests, with results presented as mean ± standard deviation. For non-normally distributed variables, the Mann–Whitney U test was employed. A *p*-value <0.05 was considered indicative of statistical significance. All statistical analyses were conducted using R (v4.2.3), leveraging the packages “tidymodels,” “glmnet,” “kernelshap,” and “shapviz.”

## Results

### Patient characteristics

**Figure 1:**
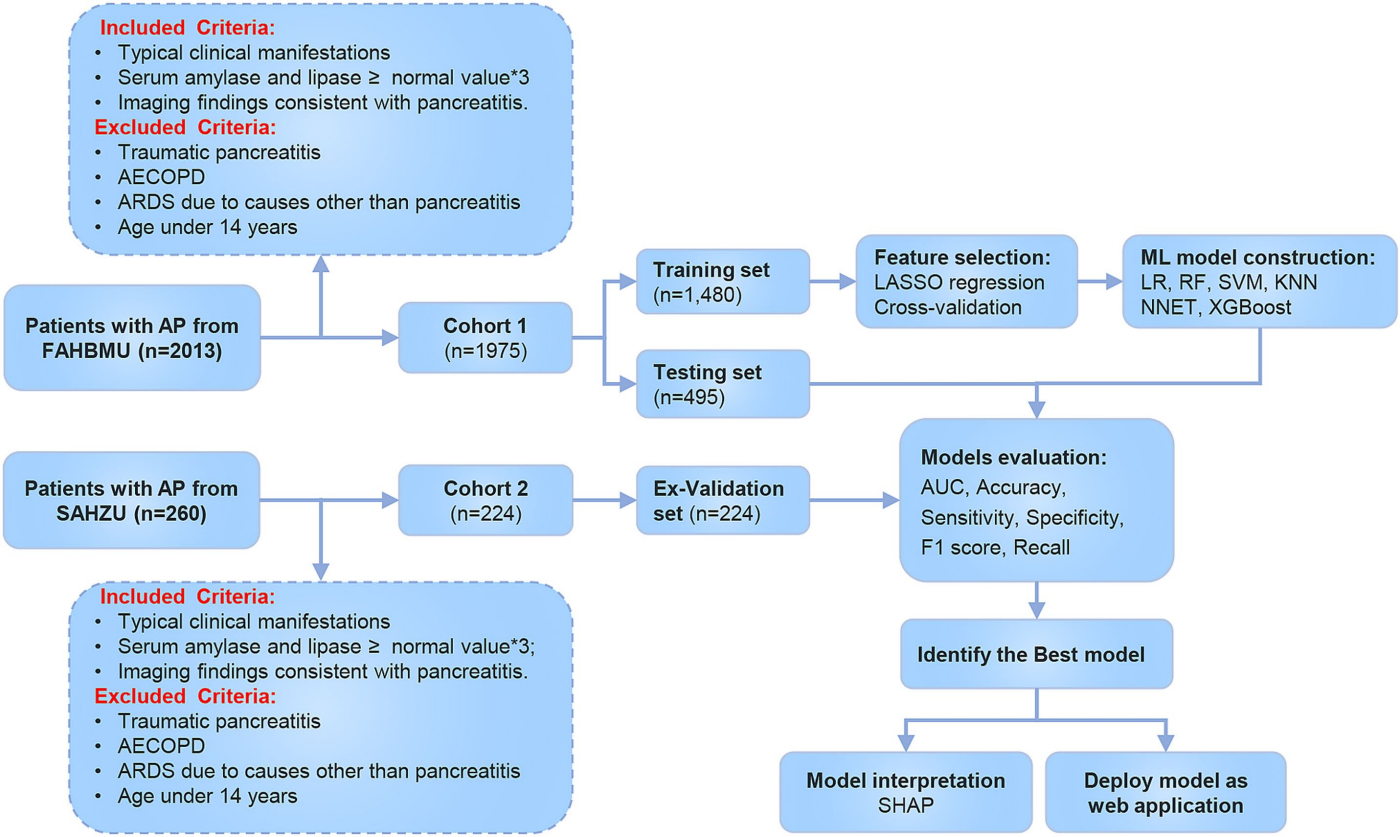
Screening and research process for acute pancreatitis-related lung injury.

The flowchart of the study is shown in Figure 1. A total of 1,975 patients with AP from the First Affiliated Hospital of Bengbu Medical University were included in the study. The patients were randomly assigned to a training set (1,480 cases) and a validation set (495 cases) in a 3:1 ratio (Table 1). In the training set, 480 cases (32.43%) of APALI were identified, consisting of 268 males (55.83%) and 212 females (44.17%). Among the 1,000 cases (67.57%) without APALI, 566 were male (56.60%) and 434 were female (43.40%) (Table 2). The gender distribution did not differ significantly between the two groups. Table 2 and Supplementary Table S1 summarize the baseline characteristics of the training and testing set. Compared to the non-APALI group, patients in the APALI group were significantly younger (50 [38, 66] vs. 47 [35, 65]) (*p* < 0.05). In addition, the APALI group showed significantly higher levels of calcium ions, neutrophil count, lymphocyte count, lactate, BMI, pulse, blood glucose, CRP, and other markers. In comparison, albumin levels were notably lower (*p* < 0.05). More importantly, levels of NLR, CRP, Triglyceride-Glucose Index, PLR, NPR, NAR, blood amylase, and urinary amylase were significantly higher in the APALI group compared to the non-APALI group (*p* < 0.05). The APALI group also had a higher prevalence of pleural effusion. In the testing set of 495 patients, 161 (32.53%) had APALI, with 93 males (57.76%) and 68 females (42.24%). In the non-APALI group (*n* = 334), 182 were males (54.49%) and 152 were females (45.51%). The gender distribution was similar to the training set, with consistent findings in the validation set (Supplementary Table S1).

**Table 1:** The characteristics of patients with APALI.

Characteristic	Training set (*N* = 1,480) ^a^	Testing set (*N* = 495) ^a^	External validation set (*N* = 224) ^a^
Outcome			
Non-APALI	1,000 (68.57%)	334 (67.47%)	151 (67.41%)
APALI	480 (32.43%)	161 (32.53%)	73 (32.59%)
Gender			
Male	834 (56.35%)	275 (55.56%)	154 (68.75%)
Female	646 (43.65%)	220 (44.44%)	70 (31.25%)
Age (years)	49 (37, 66)	49 (38, 65)	53 (41, 66)
Respiratory rate (cpm)	21.20 (2.81)	21.07 (2.70)	20.71 (13.03)
Ca^2+^ (mmol/l)	1.07 (1.00, 1.15)	1.08 (1.01, 1.15)	1.76 (1.26, 2.07)
Platelet (×10^9/l)	206.0 (159.25, 256.0)	194.0 (150.25, 255.0)	199.0 (159.0, 269.5)
Neutrophil (×10^9/l)	9.84 (6.95, 13.79)	9.78 (6.40, 13.42)	11.20 (7.90, 16.01)
Lymphocyte (×10^9/l)	1.12 (0.79, 1.57)	1.15 (0.78, 1.62)	1.05 (0.75, 1.51)
Globulin (g/l)	33.50 (29.7, 37.5)	33.15 (28.98, 37.43)	31.70 (28.83, 35.23)
Albumin (g/l)	39.30 (34.6, 43.0)	39.15 (35.0, 43.2)	35.90 (31.0, 40.5)
Total cholesterol (mmol/l)	4.01 (3.13, 5.46)	3.95 (3.11, 5.35)	3.84 (2.87, 5.22)
Pleural effusion	488 (32.97%)	177 (35.76%)	67 (29.91%)
CTSI			
A	42 (2.84%)	12 (2.42%)	44 (19.64%)
B	758 (51.22%)	256 (51.72%)	79 (35.27%)
C	618 (41.75%)	213 (43.03%)	51 (22.77%)
D	62 (4.19%)	14 (2.83%)	14 (6.25%)
E	0 (0%)	0 (0%)	36 (16.07%)
Lactate (mmol/l)	1.28 (0.84, 2.23)	1.30 (0.84, 2.21)	1.90 (1.08, 2.75)
BMI	25.15 (3.09)	25.08 (3.15)	24.55 (3.52)
Temperature (°C)	36.88 (0.54)	36.84 (0.49)	36.89 (1.09)
Pulse (bpm)	92.95 (82.0, 100.0)	92.95 (81.0, 99.0)	92.95 (84.0, 101.0)
SBP (mmHg)	133.51 (126.0, 140.0)	133.51 (125.0, 139.0)	133.51 (127.0, 141.0)
DBP (mmHg)	79.19 (76.0, 87.0)	79.19 (76.0, 88.0)	79.19 (76.0, 86.0)
RDW	13.69 (1.40)	13.65 (1.29)	13.63 (1.25)
Blood glucose (mmol/l)	7.82 (6.15, 11.23)	7.83 (6.01, 10.96)	7.33 (5.85, 9.82)
NLR	8.91 (5.15, 14.23)	8.65 (5.11, 13.67)	11.07 (6.86, 15.75)
C-reactive protein (mg/l)	46.92 (12.29, 90.0)	45.00 (11.18, 90.0)	137.00 (35.0, 229.90)
TyG	3.44 (3.07, 3.98)	3.42 (3.01, 3.96)	2.71 (2.27, 3.18)
PLR	179.08 (126.49, 260.76)	180.75 (123.17, 245.23)	180.61 (110.37, 283.96)
NPR	0.05 (0.03, 0.07)	0.05 (0.03, 0.07)	0.05 (0.04, 0.08)
NAR	0.26 (0.18, 0.37)	0.25 (0.17, 0.36)	0.32 (0.22, 0.46)
Amylase (ln) ^b^	5.37 (4.39, 6.42)	5.51 (4.44, 6.50)	5.63 (4.63, 6.48)
Urinary Amylase (ln) ^b^	6.61 (5.51, 8.30)	6.86 (5.82, 8.47)	5.96 (5.25, 6.96)
Triglyceride (ln) ^b^	0.52 (0.08, 1.33)	0.52 (−0.03, 1.28)	0.46 (0.12, 1.05)
Procalcitonin (ng/ml)	−0.48 (−2.12, 0.66)	−0.63 (−2.21, 0.66)	0.75 (0.23, 1.72)
SII (ln) ^b^	10.53 (9.98, 11.03)	10.47 (9.95, 10.92)	7.75 (7.13, 8.26)

a Mean (SD), Median (Q1, Q3); *n* (%).

b ln, Natural logarithm.

CTSI, CT Severity Index; BMI, Body Mass Index; SBP, Systolic Blood Pressure; DBP, Diastolic Blood Pressure; RDW, Red Cell Distribution Width; NLR, Neutrophil-to-Lymphocyte Ratio; TyG, Triglyceride-Glucose Index; PLR, Platelet-to-Lymphocyte Ratio; NPR, Neutrophil-to-Platelet Ratio; NAR, Neutrophil-to-Albumin Ratio; SII, Systemic Inflammation Index.

**Table 2:** The characteristics of patients with acute pancreatitis in the training set.

Characteristic	Non-APALI (*N* = 1,000) ^a^	APALI (*N* = 480) ^a^	*P*-value ^b^
Gender			0.80
Male	566 (56.60%)	268 (55.83%)	
Age (years)	50 (38, 66)	47 (35, 65)	0.02
Respiratory rate (cpm)	21.08 (2.76)	21.48 (2.89)	0.002
Ca^2+^ (mmol/l)	1.07 (1.00, 1.13)	1.10 (1.00, 1.21)	<0.001
Platelet (×10^9/l)	204.0 (157.75, 253.0)	211.0 (162.0, 263.75)	0.30
Neutrophil (×10^9/l)	8.84 (5.88, 12.07)	12.80 (9.76, 16.90)	<0.001
Lymphocyte (×10^9/l)	1.10 (0.78, 1.52)	1.16 (0.82, 1.67)	0.05
Globulin (g/l)	33.60 (29.80, 37.70)	33.40 (29.50, 37.20)	0.80
Albumin (g/l)	40.30 (35.70, 43.45)	37.10 (32.60, 41.50)	<0.001
Total cholesterol (mmol/l)	4.05 (3.24, 5.46)	3.94 (2.94, 5.46)	0.06
Pleural Effusion	262 (26.20%)	226 (47.08%)	<0.001
CTSI			<0.001
A	37 (3.70%)	5 (1.04%)	
B	606 (60.60%)	152 (31.67%)	
C	338 (33.80%)	280 (58.33%)	
D	19 (1.90%)	43 (8.96%)	
Lactate (mmol/l)	1.15 (0.80, 2.01)	1.56 (0.94, 2.90)	<0.001
BMI	24.52 (3.08)	26.44 (2.70)	<0.001
Temperature (°C)	36.85 (0.51)	36.96 (0.58)	<0.001
Pulse (bpm)	92.95 (80.0, 98.0)	92.95 (87.0, 102.0)	<0.001
SBP (mmHg)	133.51 (125.0, 141.0)	133.51 (126.75, 138.0)	0.80
DBP (mmHg)	79.19 (76.0, 87.0)	79.19 (76.0, 85.0)	0.20
RDW	13.63 (1.41)	13.81 (1.37)	<0.001
Blood glucose (mmol/l)	7.68 (6.11, 10.76)	8.22 (6.31, 11.96)	0.019
NLR	8.03 (4.42, 13.06)	10.63 (7.31, 16.65)	<0.001
C-reactive protein (mg/l)	27.65 (7.83, 76.18)	90.0 (48.72, 90.0)	<0.001
TyG	3.44 (3.08, 3.93)	3.44 (3.03, 4.09)	0.80
PLR	181.0 (128.67, 264.01)	174.59 (124.19, 251.53)	0.30
NPR	0.04 (0.03, 0.06)	0.06 (0.04, 0.09)	<0.001
NAR	0.23 (0.15, 0.31)	0.35 (0.25, 0.46)	<0.001
Amylase (ln) ^c^	5.51 (4.45, 6.55)	5.05 (4.28, 6.12)	<0.001
Urinary amylase (ln) ^c^	6.70 (5.59, 8.36)	6.42 (5.34, 8.04)	0.004
Triglyceride (ln) ^c^	0.48 (0.07, 1.28)	0.62 (0.08, 1.38)	0.13
Procalcitonin (ng/ml)	−0.40 (−2.21, 0.66)	−0.56 (−1.97, 0.66)	0.40
SII (ln) ^c^	10.53 (10.00, 11.02)	10.52 (9.97, 11.04)	>0.90

a Mean (SD), Median (Q1, Q3); *n* (%).

b Pearson’s Chi-squared test; Wilcoxon rank sum test.

c ln, Natural logarithm.

CTSI, CT Severity Index; BMI, Body Mass Index; SBP, Systolic Blood Pressure; DBP, Diastolic Blood Pressure; RDW, Red Cell Distribution Width; NLR, Neutrophil-to-Lymphocyte Ratio; TyG, Triglyceride-Glucose Index; PLR, Platelet-to-Lymphocyte Ratio; NPR, Neutrophil-to-Platelet Ratio; NAR, Neutrophil-to-Albumin Ratio; SII, Systemic Inflammation Index.

For the external validation, 224 additional patients with AP were recruited from the Second Affiliated Hospital of Zhejiang University (Supplementary Table S2). The cohort included 151 cases in the non-APALI group (70.20% males and 29.80%females) and 73 cases in the APALI group (66.75% males and 34.25%females). The characteristics of this cohort were consistent with those of the training set (*p* < 0.05). Compared to the training and testing set, patients in the external validation set were significantly older and had markedly higher levels of Ca^2+^, neutrophil count, lymphocyte count, lactate, and CRP further supporting the external validation cohort better represents a wide range of clinical scenarios, enhancing the generalizability and reliability of the study findings.

### Feature selection

**Figure 2:**
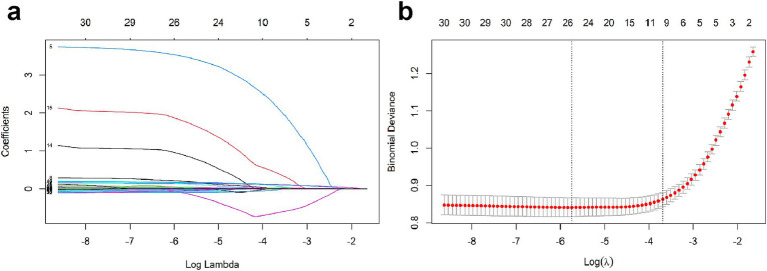
**(a)** LASSO coefficient profiles for texture features. **(b)** Selection of tuning parameter λ via 10-fold cross-validation in LASSO penalized logistic regression.

We performed LASSO regression analysis combined with cross-validation to identify potential risk factors associated with APALI. LASSO regression incorporates a penalty term to mitigate multicollinearity, optimize variable selection, and enhance model stability and interpretability. After the LASSO regression selection, 25 candidate predictors were determined, including Age, Respiratory Rate, Calcium ions, Platelet, Neutrophil count, Lymphocyte, Globulin, Cholesterol, Pleural effusion, CT grade, Lactate, BMI, Temperature, Pulse, SBP, DBP, Red blood cell width, Blood Glucose, NLR, CRP, and NAR (Figures 2a,b). These variables were used as potential predictors in development of the machine learning model. Six machine learning methods-logistic regression, random forest, XGBoost, SVM, KNN, NNET were applied to construct the risk models.

### Model evaluation in the training/testing set and the external validation set

**Figure 3:**
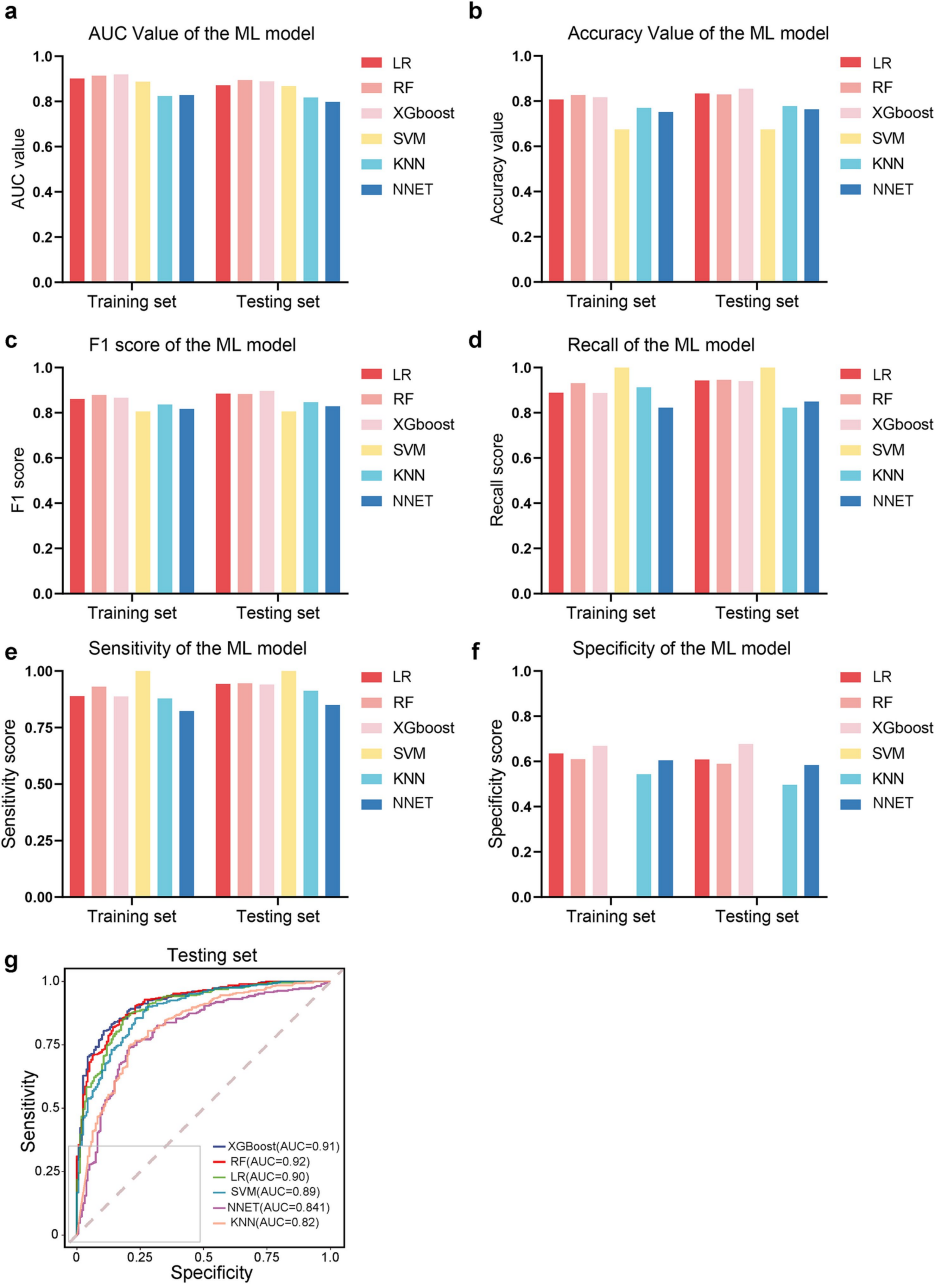
Performance comparison of machine learning models. **(a–f)** Bar plots or metrics distributions (AUC, accuracy, F1 score, recall, sensitivity, specificity) across models. **(g)** Receiver operating characteristic (ROC) curves for each model in the testing set.

The comparative performance of all models is summarized in Tables 3, 4 and Figures 3a–f. After evaluating six machine learning algorithms on both training and testing datasets, XGBoost was selected as the primary model due to its superior performance on the test set, achieving an AUC of 0.91 (95% CI: 0.89–0.94), an F1 score of 0.90 (95% CI: 0.88–0.92), a sensitivity of 0.95 (95% CI: 0.92–0.97), and a specificity of 0.68 (95% CI: 0.61–0.75). The random forest (RF) model attained the highest AUC of 0.92 (95% CI: 0.89–0.94) and was retained as a secondary tool for reassessing uncertain cases. Both tree-based models outperformed linear models (e.g., LR: AUC = 0.90 [95% CI: 0.87–0.93], F1 = 0.88 [95% CI: 0.86–0.91]) and (NNET: AUC = 0.81 [95% CI: 0.77–0.85], F1 = 0.82 [95% CI: 0.78–0.85]) across all metrics. The comparable recall (XGBoost: 0.95 [95% CI: 0.92–0.97]; RF: 0.93–0.97) and F1 scores (XGBoost: 0.90; RF: 0.89) further demonstrate their robust feature-learning capabilities. Overall, XGBoost appears more suitable for clinical applications, whereas RF may serve as a complementary tool for exploratory or confirmatory analyses. The ROC curves for all models in the test set are presented in Figure 3g.

**Table 3:** Comparison of the performance of the six models in training set.

Model	Training set					
	Accuracy	AUC	F1	Recall	Sensitivity	Specificity
KNN	0.79 (0.77, 0.81)	0.84 (0.82, 0.86)	0.85 (0.84, 0.87)	0.91 (0.89, 0.93)	0.91 (0.89, 0.93)	0.54 (0.50, 0.58)
LR	0.81 (0.79, 0.83)	0.87 (0.86, 0.89)	0.87 (0.85, 0.88)	0.90 (0.88, 0.92)	0.90 (0.88, 0.92)	0.63 (0.59, 0.67)
NNET	0.79 (0.77, 0.81)	0.86 (0.84, 0.88)	0.85 (0.83, 0.86)	0.87 (0.85, 0.89)	0.87 (0.85, 0.89)	0.63 (0.59, 0.67)
RF	0.83 (0.81, 0.84)	0.90 (0.88, 0.91)	0.88 (0.86, 0.89)	0.94 (0.92, 0.95)	0.94 (0.92, 0.95)	0.60 (0.55, 0.64)
SVM	0.79 (0.77, 0.81)	0.87 (0.85, 0.88)	0.84 (0.82, 0.85)	0.80 (0.78, 0.83)	0.80 (0.78, 0.83)	0.75 (0.71, 0.79)
XGboost	0.82 (0.80, 0.84)	0.90 (0.88, 0.91)	0.87 (0.85, 0.89)	0.90 (0.88, 0.91)	0.90 (0.88, 0.91)	0.66 (0.62, 0.71)

LR, Logistic regression; RF, Random Forest; XGBoost, Extreme Gradient Boosting; SVC, Support vector Classifier; KNN, k-nearest neighbor; NNET, Neural Network.

**Table 4:** Comparison of the performance of the six models in testing set.

Model	Testing set					
	Accuracy	AUC	F1	Recall	Sensitivity	Specificity
KNN	0.78 (0.74, 0.81)	0.82 (0.78, 0.86)	0.85 (0.82, 0.87)	0.91 (0.88, 0.94)	0.91 (0.88, 0.94)	0.50 (0.42, 0.57)
LR	0.83 (0.80, 0.86)	0.90 (0.87, 0.93)	0.88 (0.86, 0.91)	0.94 (0.92, 0.97)	0.94 (0.92, 0.97)	0.61 (0.53, 0.68)
NNET	0.74 (0.70, 0.78)	0.81 (0.77, 0.85)	0.82 (0.78, 0.85)	0.84 (0.80, 0.88)	0.84 (0.80, 0.88)	0.53 (0.45, 0.61)
RF	0.84 (0.81, 0.87)	0.92 (0.89, 0.94)	0.89 (0.87, 0.91)	0.95 (0.93, 0.97)	0.95 (0.93, 0.97)	0.62 (0.55, 0.69)
SVM	0.82 (0.79, 0.85)	0.89 (0.86, 0.92)	0.87 (0.84, 0.89)	0.86 (0.83, 0.90)	0.86 (0.83, 0.90)	0.74 (0.67, 0.81)
XGboost	0.86 (0.83, 0.89)	0.91 (0.89, 0.94)	0.90 (0.88, 0.92)	0.95 (0.92, 0.97)	0.95 (0.92, 0.97)	0.68 (0.61, 0.75)

LR, Logistic regression; RF, Random Forest; XGBoost, Extreme Gradient Boosting; SVC, Support vector Classifier; KNN, k-nearest neighbor; NNET, Neural Network.

To evaluate the generalizability of our model, we tested its performance on an external validation set to classify patients with APALI and non-APALI. The performance of the models is summarized in Supplementary Table S3. All models in the independent external validation set demonstrated strong discriminative performance, with ROC-AUC values consistently exceeding 0.750. Among them, the RF model exhibited statistically superior performance (DeLong’s test, *p* < 0.001). Notably, the XGBoost model also displayed competitive performance, with marginally higher metrics compared to the remaining models, including an AUROC of 0.990, accuracy (0.953), F1 score (0.900), recall (0.987), sensitivity (0.987), and specificity (0.975). The result indicated that the XGBoost and RF models exhibited good performance and clinical utility, whereas the SVM, KNN, and NNET models showed relatively lower performance Supplementary Figure S1.

### Model interpretation and variables of importance

**Figure 4:**
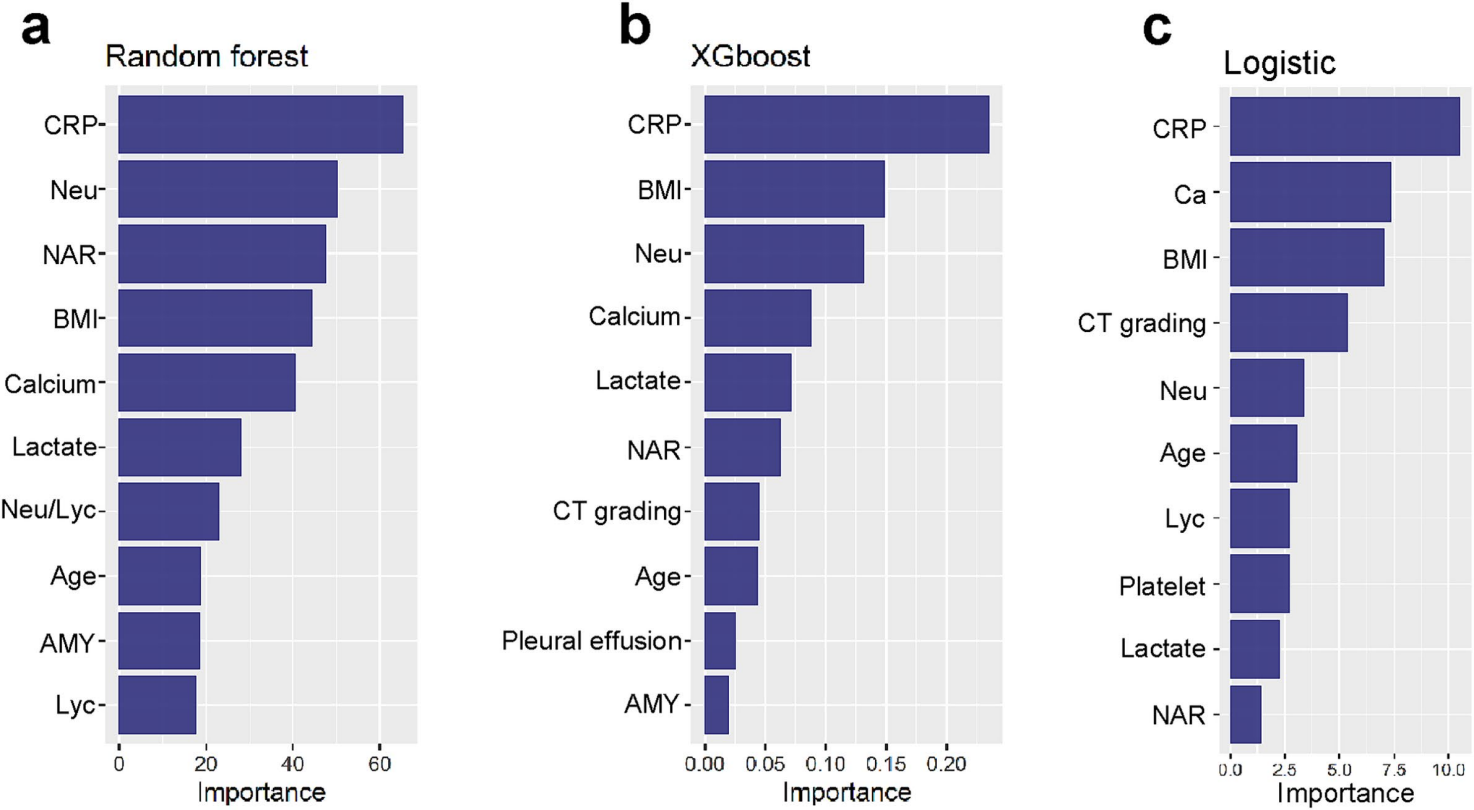
**(a–c)** Global interpretability of top-performing models (XGBoost, RF, LR) via SHAP. Summary plots rank the top 10 clinical features by mean absolute SHAP values, indicating their predictive contribution to APALI. Key features are CRP, neutrophil count, NAR, BMI, calcium ions, lactate, age, CT grade, lymphocytes (Lym), blood amylase, and pleural effusion.

Among the models evaluated, Random Forest, XGBoost, and Logistic Regression demonstrated favorable predictive performance. To enhance our understanding of model decisions, we utilized Shapley Additive Explanations (SHAP), which provides insights into the significance of individual features and their interactions within the model. SHAP values quantify the contribution of each clinical variable, with positive values reflecting an increased probability of developing APALI, and negative values suggesting a decreased likelihood. The global interpretability of these models was visualized using the SHAP summary plot, which ranked the importance of variables across clinical outcomes. As shown in Figure 4, the top 10 most influential features were identified. The variables significantly impacting the model’s predictions included CRP, neutrophil count, NAR, BMI, calcium ion levels, lactate, age, CT grade, Lym, blood amylase, and pleural effusion.

**Figure 5:**
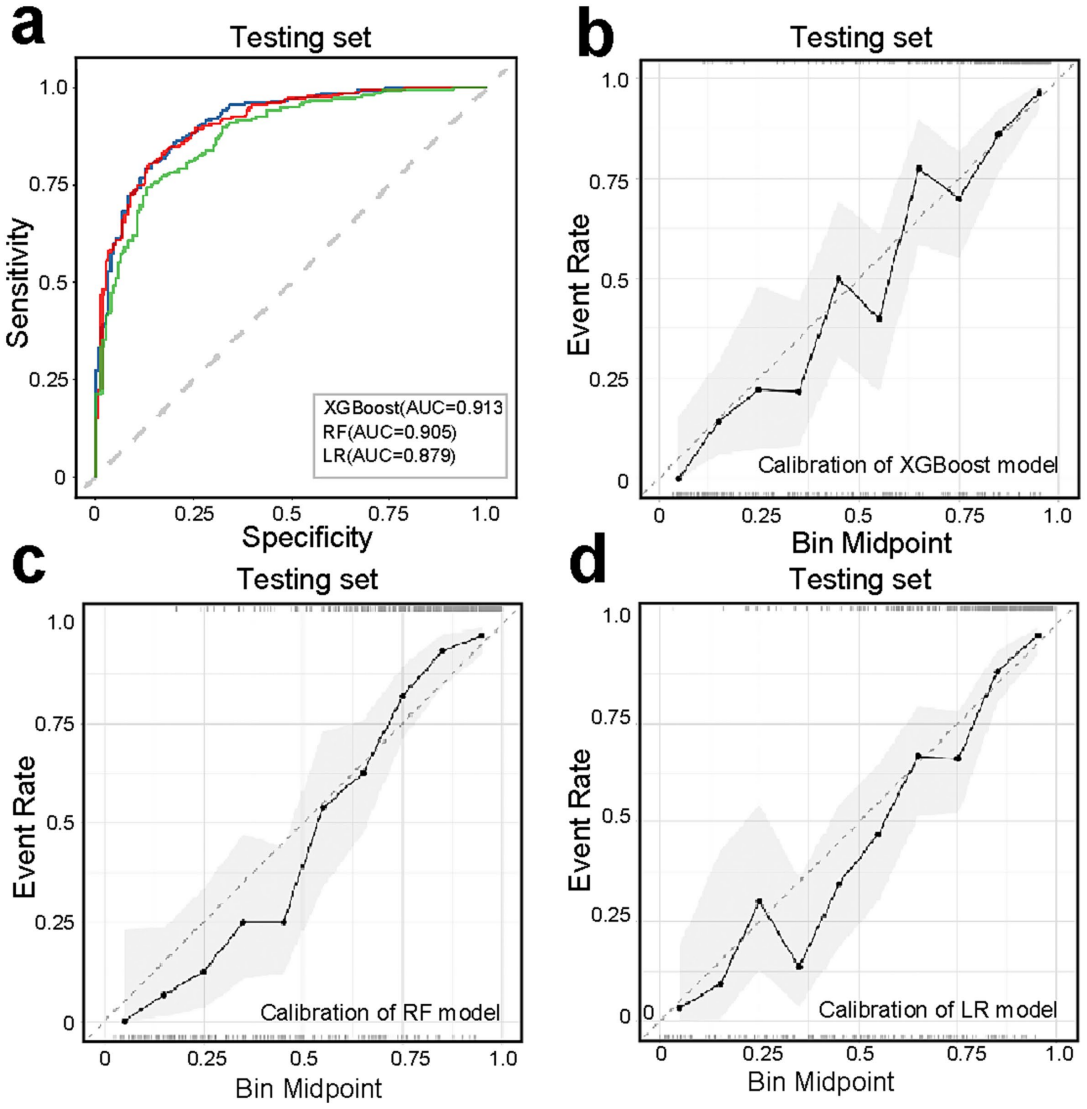
Performance evaluation of simplified models (XGBoost, RF, LR) using six key predictors (CRP, BMI, neutrophil count, calcium ions, lactate, NAR). **(a)** ROC curves demonstrating maintained predictive accuracy for XGBoost, RF, LR model despite feature reduction. **(b–d)** Calibration curves assessing agreement between predicted probabilities and observed outcomes, with closer-to-diagonal curves indicating better reliability.

**Figure 6:**
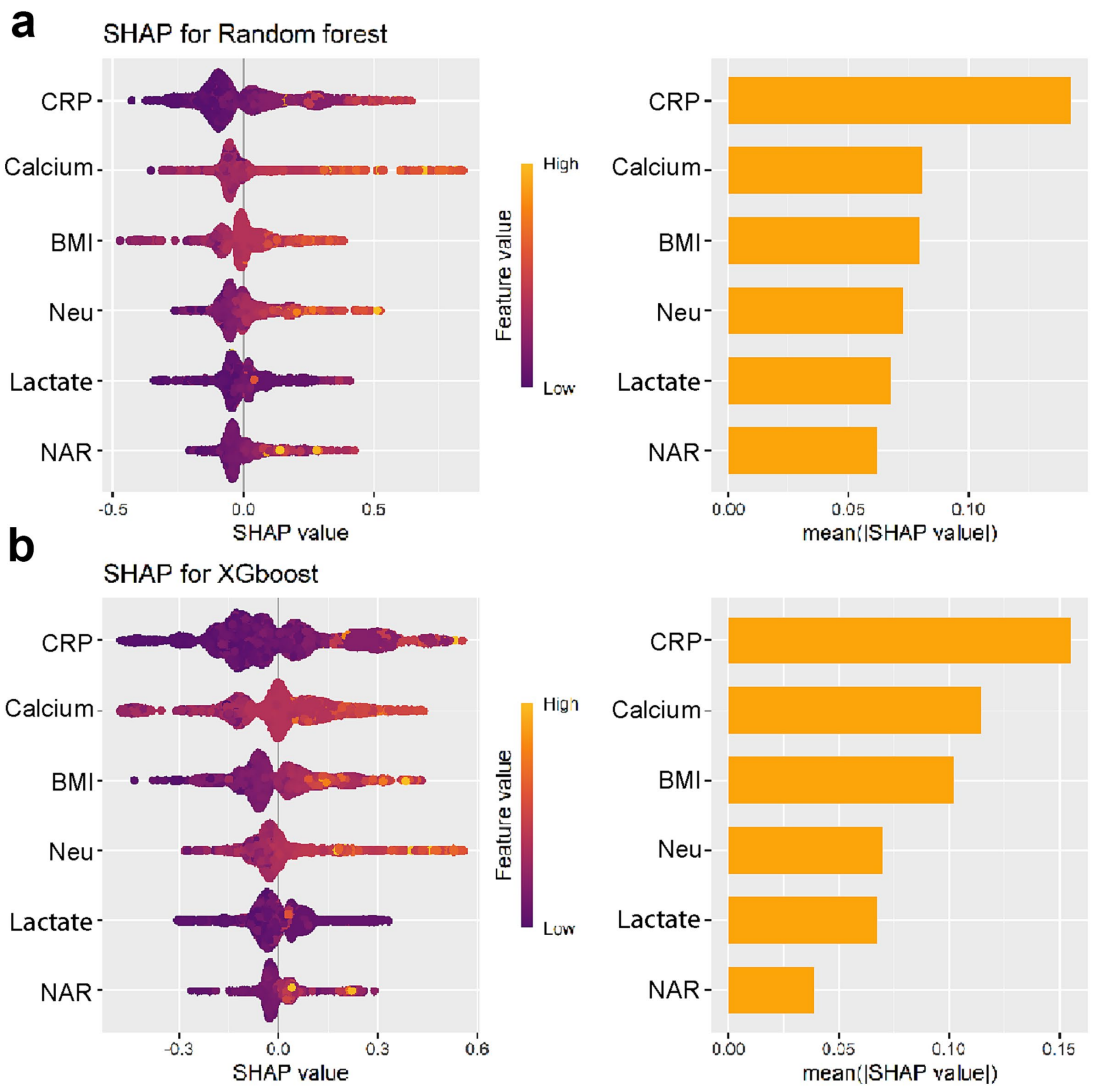
Global interpretability of the simplified XGBoost **(a)** and RF **(b)** models using SHAP summary plots.

**Figure 7:**
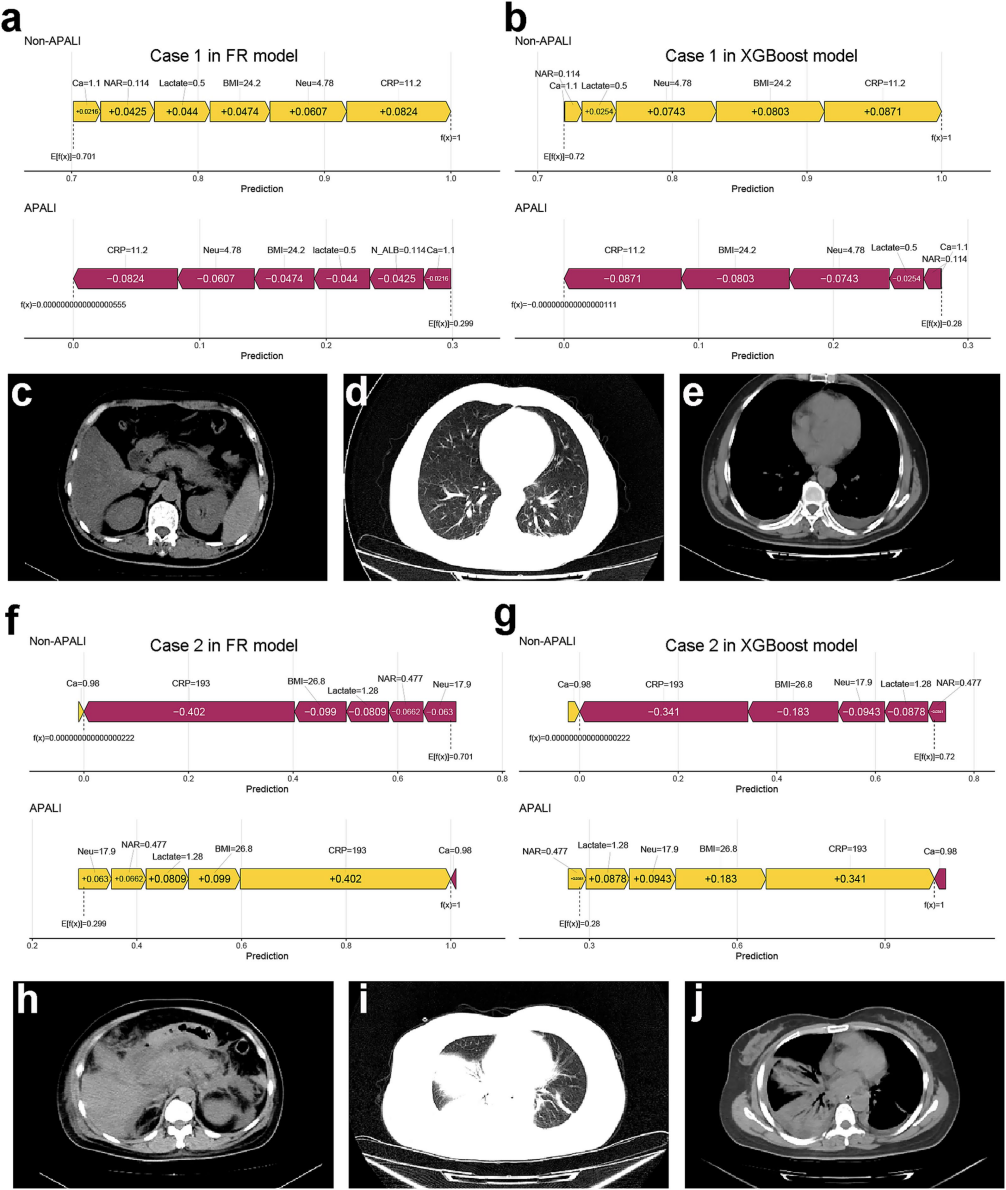
Case examples demonstrating model interpretability and clinical correlation. **(a,b)** SHAP force plots showing low-risk prediction scores for a non-APALI case. **(c–e)** Corresponding abdominal **(c)** and chest **(d,e)** CT images demonstrating absence of pulmonary abnormalities. **(f,g)** SHAP force plots of an APALI case with high-risk prediction. **(h–j)** Confirmatory CT findings showing abdominal **(h)** and chest **(i,j)** imaging with characteristic lung consolidation and pleural effusion, validating the model’s predictive accuracy.

To enhance model simplicity while maintaining predictive performance, six variables—CRP, BMI, Neu, Calcium, Lactate, and NAR—were selected based on variable importance rankings and clinical relevance for model retraining. As illustrated in Figures 5a–d, the receiver operating characteristic (ROC) curves and calibration plots demonstrated that both the random forest (RF) and XGBoost models exhibited strong predictive performance. Furthermore, decision curve analysis (DCA) indicated that both models provided greater net clinical benefit across a wide range of clinically relevant threshold probabilities compared to the “treat-all” and “treat-none” strategies (Supplementary Figure S2). Additionally, the global interpretability of the XGBoost and RF model, along with the six most influential features, is depicted in the SHAP summary plot (Figures 6a,b). To further illustrate the model’s interpretability, we present two representative cases. The first cases describe patients who did not develop APALI and had low SHAP prediction scores (Figures 7a,b). The abdominal CT (Figure 7c) and chest CT (Figures 7d,e) demonstrated that this AP patient did not have signs of lung injury. In contrast, the second case involved a patient diagnosed with APALI, showing a high SHAP prediction score (Figures 7f,g). And the abdominal CT (Figure 7h) and chest CT (Figures 7i,j) demonstrated that this AP patient had obvious lung consolidation with associated pleural effusion. All of these results proved the accuracy of the prognostic prediction system.

### Application of the model

**Figure 8:**
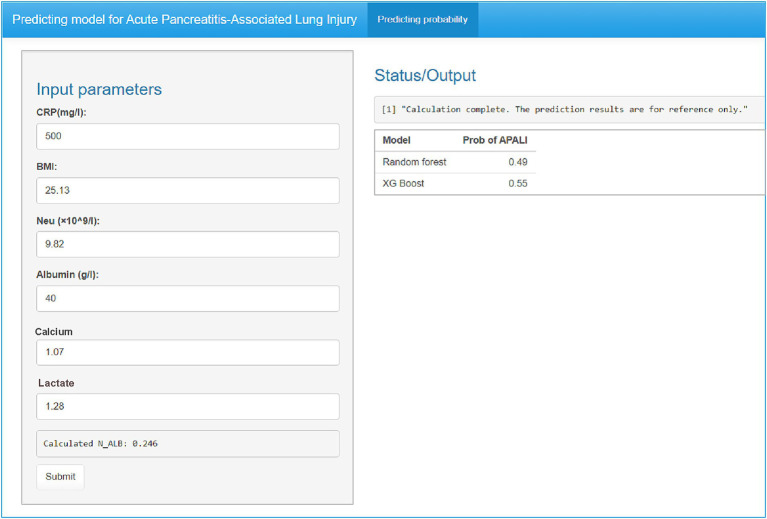
Web-based clinical decision support tool for APALI risk prediction. Screenshot of the interactive interface showing input parameters (CRP, BMI, neutrophil count/albumin, calcium, lactate) and real-time risk calculation. Example output displaying predicted APALI probability with interpretative guidance. The tool is publicly available at: https://yyiyis.shinyapps.io/APALI/.

To enhance the convenience and practice utility of the developed model, we created a web-based tool to facilitate clinicians in clinical decision-making (accessible at: https://yyiyis.shinyapps.io/APALI/). Using this tool, clinicians can input key clinical data, such as BMI, Neu (neutrophil), ALB (albumin), CRP, and calcium ion levels, to predict likelihood of APALI in patients with AP (Figure 8).

## Discussion

This study is the first and most comprehensive to apply machine learning methods to develop a model for predicting the occurrence of acute pancreatitis-associated lung injury (APALI). The RF and XGBoost models outperformed four other machine learning algorithms in the validation set, demonstrating superior AUC, accuracy, and F1 score, and exhibiting strong discriminatory power and calibration performance. We identified CRP, BMI, neutrophil count, calcium ions, lactate, and NAR as the most important independent predictors for APALI. Additionally, we developed a web-based tool to enhance the model’s convenience and practical utility. This study may facilitate the early detection of lung injury in AP and support healthcare teams in delivering targeted interventions, accelerating recovery, and improving quality of life.

Currently, there are limited studies on the prediction for ALI in AP. Jayanta Samanta identified that IL-6 and IL-8 could predict the development of ALI in AP and may serve as a composite biomarker (10). Lawrence Owusu proposed that *γ*-enolase could serve as an early indicator of lung tissue damage, even before significant histopathological injury is evident (22). Although the efficacy of these biomarkers has been partially validated, their complexity and limited availability may restrict their clinical applicability. Mengyu Jia developed a predictive model incorporating routine clinical data, such as diabetes, oxygen supplementation, neutrophil count, and D-dimer levels, and visualized the model using a nomogram (11). While this study based on relatively small cohorts (91 cases) which may undermine the stability of the model and restrict its practical applicability and suggest that machine learning models may outperform traditional manual scoring systems. Fei et al. compared artificial neural networks with logistic regression in predicting acute ALI among 217 patients with SAP. The ANN model achieved a sensitivity of 87.5%, specificity of 83.3%, and overall accuracy of 84.43%, significantly outperforming logistic regression (23). Ong et al. demonstrated that ML models (XGBoost) modestly outperformed conventional statistical methods in predicting poor post-ICU outcomes for mechanically ventilated patients, albeit with limited sensitivity at high specificity thresholds (17). These results suggest that ML offer superior early-warning capabilities for AP-related disease. Therefore, we conducted a retrospective study aimed at identifying valuable risk factors for APALI via six machine learning. The result revealed that The XGBoost and Random Forest (RF) models demonstrated the best predictive performance, achieving the highest AUC, along with higher accuracy, F1 score, and recall in the testing set. And we identified six key risk factors (CRP, BMI, neutrophil count, calcium ions, lactate, and NAR), which may predict the occurrence of APALI in the early phase of the disease. In our study, although both KNN and logistic regression models performed reasonably well, their overall predictive performance was inferior to that of the random forest and XGBoost models. Compared to previous studies, our research is based on a larger-scale population dataset and demonstrates significant advantages across multiple key predictive indicators. The model’s predictive performance has notably improved, particularly in terms of accuracy, sensitivity, and specificity, achieving high levels in these areas. These results indicate that our model not only holds strong clinical application value but also has considerable potential for widespread implementation, offering robust support for early disease prediction and intervention in broader clinical practices.

This study demonstrates that the neutrophil-to-albumin ratio (NAR) is a significant predictor of APALI, a relationship seldom explored in previous research. Neutrophils play a central role in APALI pathogenesis by releasing inflammatory mediators—such as myeloperoxidase (MPO) (24, 25), matrix metalloproteinases (MMP-8 and MMP-9), neutrophil elastase, and other proteolytic enzymes—that collectively drive systemic inflammation and tissue damage. Albumin reflects nutritional and hepatic status and declines during inflammation, infection, and organ dysfunction (26, 27). By integrating inflammatory burden and nutritional status, NAR has recently emerged as a robust prognostic biomarker across multiple clinical contexts. Elevated NAR predicts poor outcomes in sepsis, cardiovascular disease, and various malignancies, reflecting its strong association with systemic inflammation and organ failure (28). For example, in sepsis, high NAR correlates with increased mortality and organ dysfunction (29). Similar patterns appear in colorectal, gastric, and lung cancers, likely reflecting a proinflammatory tumor microenvironment (30). Furthermore, elevated NAR has been linked with adverse long-term outcomes in cardiovascular disease, including higher risks of heart failure and cardiac events (31–34).

Large-scale population studies further support NAR’s prognostic value, as well as that of its variant, the neutrophil-to-prealbumin ratio. Feng et al. reported that each 10-point increase in the Life’s Crucial 9 score corresponded to a 28% reduction in COPD odds, with NAR mediating 4.8% of the effect (35). Han et al. identified a J-shaped relationship between NAR and all-cause or cardiovascular mortality in CKD, partially mediated by eGFR (36). Li et al. found that CKD patients with cardiovascular disease and elevated NPAR had significantly increased mortality risks (37). Ma et al. demonstrated a linear association between log-transformed NPAR and albuminuria, and Gao et al. identified NAR as a key predictor of poor outcomes after endovascular stroke therapy (38, 39). In our study, NAR ranked third in importance in the random forest model—after CRP and neutrophil count—and was strongly and positively correlated with APALI. Given its simplicity and predictive strength, NAR may serve as an efficient and early biomarker for identifying patients at high risk of APALI.

Lactate levels reflect tissue hypoxia, metabolic disturbances, and inflammatory responses, all of which are prevalent in AP (40). Lactate accumulation arises from microcirculatory dysfunction and tissue hypoxia, central to the pathogenesis of acute lung injury and ARDS (40). Studies have shown that persistently elevated lactate levels are associated with poor outcomes, complications such as ARDS, and increased mortality in AP (41). Therefore, lactate monitoring is essential for prognostication and guiding therapeutic interventions, including fluid resuscitation and oxygen therapy (42, 43). The relationship between BMI and AP, as well as its associated lung injuries, such as ARDS, represents an important area of research (44, 45). Our study showed that BMI was ranked highly in both the Random Forest and XGBoost model. Obesity negatively impacts pancreatic health by disrupting fat metabolism, inducing insulin resistance, and promoting the release of pro-inflammatory cytokines. These factors contribute to systemic inflammation in patients with AP, increasing the risk of lung injury (44–46). An elevated BMI may further amplify inflammatory responses, as excess adipose tissue releases inflammatory mediators that worsen pancreatic damage and contribute to pulmonary complications (47). Therefore, a high BMI can serve as a critical early warning indicator for pulmonary injury and its progression in severe AP, playing a crucial role in the early identification and treatment of pulmonary complications.

We employed Shapley Additive Explanations (SHAP) to interpret the model and identify key clinical features influencing predictions. The SHAP values indicated that CRP, BMI, neutrophil count, calcium levels, lactate, and NAR were the most significant predictors of acute lung injury. Notably, SHAP visualizations provided clarity on the contribution of each variable and highlighted feature interactions, offering valuable insights into the underlying mechanisms of acute lung injury in AP. These findings support the utility of machine learning models in predicting the risk of acute lung injury in AP patients. By identifying key clinical features such as NAR, lactate, and BMI, our model could aid clinicians in early detection and personalized intervention. This predictive capability may facilitate timely therapies to reduce systemic inflammation and prevent progression to acute respiratory distress syndrome (ARDS). Despite strong predictive performance, real-world use of the APALI model faces three main challenges. First, technical integration: hospitals with fragmented IT systems may need middleware to support real-time use, even though the model relies on structured EHR data. Second, workflow compatibility: clinician trust requires explainability (e.g., SHAP plots) and smooth integration into systems like order entry. Third, scalability: retraining may be needed to adapt to local case-mix differences.

This study has several limitations. As a retrospective case–control study, it may be affected by selection bias, and some patients may have received treatment before laboratory data were collected, potentially influencing the results. Although we employed rigorous methodology, the external validation yielded exceptionally high performance. While we applied strict data separation and anti-leakage procedures, this result may reflect specific characteristics of the external dataset, such as small sample size, case homogeneity, and a higher proportion of critically ill patients from a tertiary referral center. Therefore, this finding should be interpreted with caution. To enhance the accuracy, generalizability, and clinical applicability of the model, future studies should focus on large-sample, multi-center prospective research, incorporate real-time clinical data, and minimize treatment-related biases.

## Conclusion

In this study, we developed a clinical prediction model for the early identification of lung injury in patients with AP by utilizing machine learning algorithms to analyze clinical data. The model exhibited robust predictive performance, validated through external testing and individual assessments, underscoring its clinical utility for timely interventions in AP-associated lung injury. Additionally, we created a web-based calculator to facilitate the model’s application in clinical practice, enabling healthcare professionals to make faster decisions and potentially improve patient outcomes. This tool represents a valuable resource for clinicians managing AP, ensuring timely and appropriate care for patients at high risk of lung injury. Future work will focus on enhancing the model through multi-center validation, incorporating diverse clinical variables, and optimizing the tool for broader clinical application.

## Data Availability

The original contributions presented in the study are included in the article/Supplementary material, further inquiries can be directed to the corresponding authors.
