# Supplementary material for: Machine learning-based predictive model for acute pancreatitis-associated lung injury: a retrospective analysis
Source: Front Med (Lausanne). 2025 Aug 12;12:1638097. doi: 10.3389/fmed.2025.1638097 (PMC12379022; doi:10.3389/fmed.2025.1638097)
Supplement: Supplementary file 1 [file Supplementary_file_1.docx]

**Supplemental Information**

**Table S1.** Baseline characteristics of patients with acute pancreatitis (AP) in the testing set.

**Table S2.** Baseline characteristics of patients with AP in the external validation set.

**Table S3.** Comparison of the predictive performance of six models in the external validation set.

**Table S4.** Selected variables and coefficients of LASSO regression with λ_min_ = 0.00321.

**Figure S1.** Clinical predictive value of six machine learning models in the external validation set.

**Figure S2.** Decision curve analysis (DCA) of the APALI prediction models (XGBoost and RF).

| **Table S1. The characteristics of patients with AP in the testing set.** | | | |
| --- | --- | --- | --- |
| **Characteristic** | **Non-APALI (N = 334)^1^** | **APALI (N = 161)^1^** | ***P*-value**^2^ |
| **Gender** |  |  | 0.50 |
| Male (%) | 182 (54.49) | 93 (57.76) |  |
| **Age(years)** | 50(40, 66) | 48 (34, 63) | 0.018 |
| **Respiratory Rate (cpm)** | 20.88 (2.63) | 21.47 (2.83) | 0.035 |
| **Ca^2+^(mmol/l)** | 1.07 (1.01, 1.13) | 1.11 (1.03, 1.21) | <0.001 |
| **Platelet (×10^9/l)** | 196.0 (156.25, 250.0) | 190.50 (140.75, 265.5) | 0.60 |
| **Neutrophil (×10^9/l)** | 9.10 (5.84, 11.80) | 12.53 (8.49, 16.12) | <0.001 |
| **Lymphocyte (×10^9/l)** | 1.11 (0.77, 1.59) | 1.20 (0.83, 1.63) | 0.20 |
| **Globulin (g/l)** | 33.50 (29.85, 37.70) | 32.30 (28.10, 36.95) | 0.025 |
| **Albumin (g/l)** | 40.00 (35.70, 43.40) | 37.40 (32.85, 41.70) | <0.001 |
| **Total Cholesterol (mmol/l)** | 3.99 (3.29, 5.43) | 3.76 (2.96, 5.08) | 0.065 |
| **Pleural Effusion** | 91 (27.25) | 86 (53.42) | <0.001 |
| **CTSI** (%) |  |  | <0.001 |
| A | 11 (3.29) | 1 (0.62) |  |
| B | 208 (62.28) | 48 (29.81) |  |
| C | 114 (34.13) | 99 (61.49) |  |
| D | 1 (0.30) | 13 (8.08) |  |
| **Lactate (mmol/l)** | 1.17 (0.80, 2.01) | 1.67 (0.96, 3.21) | <0.001 |
| **BMI** (%) | 24.42 (3.14) | 26.46 (2.69) | <0.001 |
| **Temperature (℃)** | 36.80 (0.47) | 36.94 (0.52) | 0.018 |
| **Pulse (bpm)** | 92.95 (80.0, 96.0) | 92.95 (88.0, 103.0) | <0.001 |
| **SBP (mmHg)** | 133.51 (126.0, 140.0) | 133.51 (124.0, 137.0) | 0.50 |
| **DBP (mmHg)** | 79.19 (76.0, 88.0) | 79.19 (75.0, 85.0) | 0.13 |
| **RDW** | 13.55 (1.25) | 13.86 (1.35) | 0.005 |
| **Blood Glucose (mmol/l)** | 7.76 (5.93, 10.71) | 8.09 (6.11, 11.23) | 0.50 |
| **NLR** | 7.96 (4.75, 12.15) | 10.29 (6.72, 16.23) | <0.001 |
| **C-reactive Protein (mg/l)** | 22.30 (7.99, 66.40) | 90.00 (58.49, 90.0) | <0.001 |
| **TyG** | 3.43 (3.02, 3.97) | 3.37 (2.98, 3.88) | 0.50 |
| **PLR** | 190.51 (127.10, 253.08) | 157.62 (111.97, 221.47) | 0.02 |
| **NPR** | 0.05 (0.03, 0.06) | 0.07 (0.04, 0.10) | <0.001 |
| **NAR** | 0.23 (0.15, 0.30) | 0.33 (0.23, 0.44) | <0.001 |
| **Amylase (ln)^3^** | 5.63 (4.48, 6.60) | 5.28 (4.41, 6.25) | 0.2 |
| **Urinary Amylase (ln)^3^** | 6.98 (5.80, 8.48) | 6.66 (5.85, 8.37) | 0.4 |
| **Triglyceride (ln)^3^** | 0.48 (-0.06, 1.28) | 0.63 (0.14, 1.30) | 0.10 |
| **Procalcitonin (ng/ml)** | -0.90 (-2.30, 0.66) | -0.30 (-1.71, 0.66) | 0.002 |
| **SII (ln)^3^** | 10.52 (10.01, 10.94) | 10.35 (9.77, 10.88) | 0.04 |
| 1 Mean (SD), Median (Q1, Q3); n (%) | | | |
| 2 Pearson's Chi-squared test; Wilcoxon rank sum test; Fisher's exact test；  3 ln: Natural logarithm  **Abbreviations:** CTSI: CT Severity Index; BMI: Body Mass Index; SBP: Systolic Blood Pressure; DBP: Diastolic Blood Pressure; RDW: Red Cell Distribution Width; NLR: Neutrophil-to-Lymphocyte Ratio; TyG: Triglyceride-Glucose Index; PLR: Platelet-to-Lymphocyte Ratio; NPR: Neutrophil-to-Platelet Ratio; NAR: Neutrophil-to-Albumin Ratio; SII: Systemic Inflammation Index. | | | |

| **Table S2. The characteristics of patients with AP in external validation set.** | | | |
| --- | --- | --- | --- |
| **Characteristic** | **Non-APALI (N = 151)^1^** | **APALI (N = 73)^1^** | ***P*-value**^2^ |
| **Gender** |  |  | 0.50 |
| Male (%) | 106 (70.20) | 48 (65.75) |  |
| **Age(years)** | 55 (42, 68) | 51.00 (36, 62) | 0.03 |
| **Respiratory Rate (cpm)** | 19.18 (1.67) | 25.18 (25.23) | <0.001 |
| **Platelet (×10^9/l)** | 205.0 (160.0, 279.0) | 186.0 (148.75, 251.0) | 0.061 |
| **Neutrophil (×10^9/l)** | 9.94 (6.68, 12.52) | 17.30 (13.19, 21.22) | <0.001 |
| **Lymphocyte (×10^9/l)** | 0.98 (0.63, 1.42) | 1.25 (1.02, 1.70) | <0.001 |
| **Globulin (g/l)** | 32.20 (29.20, 36.20) | 30.80 (26.80, 34.20) | 0.03 |
| **Albumin (g/l)** | 37.05 (32.70, 41.43) | 33.20 (29.60, 36.70) | 0.001 |
| **Total Cholesterol (mmol/l)** | 3.84 (2.93, 4.90) | 3.81 (2.72, 6.08) | 0.90 |
| **Pleural Effusion** | 6 (4.64) | 60 (82.19) | <0.001 |
| **CTSI** (%) |  |  | <0.001 |
| A | 42 (27.82) | 2 (2.74) |  |
| B | 72 (47.68) | 7 (9.59) |  |
| C | 36 (23.84) | 15 (20.55) |  |
| D | 1 (0.66) | 13 (17.81) |  |
| E | 0 (0.00) | 36 (49.31) |  |
| **Lactate (mmol/l)** | 1.35 (0.90, 2.11) | 2.93 (2.39, 3.70) | <0.001 |
| **BMI** (%) | 23.51 (3.23) | 26.63 (3.16) | <0.001 |
| **Temperature (℃)** | 36.87 (1.31) | 36.93 (0.51) | 0.14 |
| **Pulse (bpm)** | 89.00 (80.50, 98.0) | 92.95 (90.0, 106.0) | 0.005 |
| **SBP (mmHg)** | 133.51 (125.0, 140.0) | 133.51 (133.0, 144.0) | 0.11 |
| **DBP (mmHg)** | 79.19 (76.0, 88.50) | 79.19 (76.0, 83.0) | 0.03 |
| **RDW** | 13.58 (1.29) | 13.73 (1.15) | 0.11 |
| **Blood Glucose (mmol/l)** | 6.95 (5.61, 8.99) | 8.82 (6.75, 12.30) | <0.001 |
| **NLR** | 9.62 (5.74, 14.72) | 13.21 (9.57, 17.45) | <0.001 |
| **C-reactive Protein (mg/l)** | 88.95 (22.55, 169.0) | 208.60 (131.50, 266.20) | <0.001 |
| **TyG** | 2.57 (2.15, 2.98) | 2.95 (2.40, 3.67) | <0.001 |
| **PLR** | 217.36 (137.56, 337.53) | 137.01 (81.64, 204.13) | <0.001 |
| **NPR** | 0.04 (0.03, 0.06) | 0.09 (0.06, 0.12) | <0.001 |
| **NAR** | 0.26 (0.17, 0.37) | 0.50 (0.40, 0.68) | <0.001 |
| **Amylase (ln)^3^** | 5.27 (4.51, 6.11) | 6.07 (5.13, 6.93) | 0.006 |
| **Urinary Amylase (ln)^3^** | 5.64 (5.13, 6.48) | 6.93 (5.83, 8.45) | <0.001 |
| **Triglyceride (ln)^3^** | 0.34 (-0.05, 0.70) | 0.79 (0.36, 1.56) | <0.001 |
| **Procalcitonin (ng/ml)** | 0.77 (0.18, 1.69) | 0.71 (0.24, 2.33) | 0.60 |
| **SII (ln)^3^** | 7.68 (6.94, 8.23) | 7.84 (7.48, 8.28) | 0.08 |
| 1 Mean (SD), Median (Q1, Q3); n (%) | | | |
| 2 Pearson's Chi-squared test; Wilcoxon rank sum test；  3 ln: Natural logarithm  **Abbreviations:** CTSI: CT Severity Index; BMI: Body Mass Index; SBP: Systolic Blood Pressure; DBP: Diastolic Blood Pressure; RDW: Red Cell Distribution Width; NLR: Neutrophil-to-Lymphocyte Ratio; TyG: Triglyceride-Glucose Index; PLR: Platelet-to-Lymphocyte Ratio; NPR: Neutrophil-to-Platelet Ratio; NAR: Neutrophil-to-Albumin Ratio; SII: Systemic Inflammation Index. | | | |

**Table S3. Comparison of the performance of the six models in external validation set.**

| **Models** | **AUC** | **Accuracy** | **F1 score** | **Recall** | **Sensitivity** | **Specificity** |
| --- | --- | --- | --- | --- | --- | --- |
| **LR** | 0.989 | 0.946 | 0.967 | 0.961 | 0.967 | 0.904 |
| **RF** | **1.000** | **0.996** | **0.993** | **0.997** | **0.993** | **1.000** |
| **XGboost** | **0.990** | **0.953** | **0.987** | **0.900** | **0.987** | **0.975** |
| **SVM** | 0.969 | 0.674 | 1.000 | 0.805 | 1.000 | 0.000 |
| **KNN** | 0.998 | 0.960 | 0.987 | 0.971 | 0.987 | 0.904 |

**Abbreviations:** LR, Logistic regression; RF, Random Forest; XGBoost, Extreme Gradient Boosting; SVC, Support vector Classifier; KNN, k-nearest neighbor;

**Table S4.** **Selected variables and coefficients of LASSO regression with λ_min_ = 0.00321**

| Variable | Estimate |
| --- | --- |
| Age | -0.0088531 |
| Breathing | -0.0075417 |
| Ca | 3.4718929 |
| Platelet | -0.0008687 |
| N | 0.1499499 |
| L | 0.1924223 |
| Globulin | 0.0081009 |
| cholesterol | -0.0361379 |
| Pleural_effusion1 | 0.1619727 |
| CT_grading1 | -0.1193935 |
| CT_grading2 | 0.8729548 |
| CT_grading3 | 1.7645778 |
| lactic_acid | 0.0461642 |
| BMI | 0.1714652 |
| temperature | 0.1393557 |
| heart_rate | -0.0028294 |
| SP | 0.0006568 |
| BP | -0.0045736 |
| Red_blood_cell_width | 0.0214212 |
| Blood_sugur | -0.0195391 |
| NL | 0.0069396 |
| CRP | 0.0180950 |
| N_ALB | 0.0224100 |
| Blood_amylase_ln | -0.0865793 |
| Urinary_amylase_ln | -0.0596785 |
| triglyceride_ln | -0.0496752 |


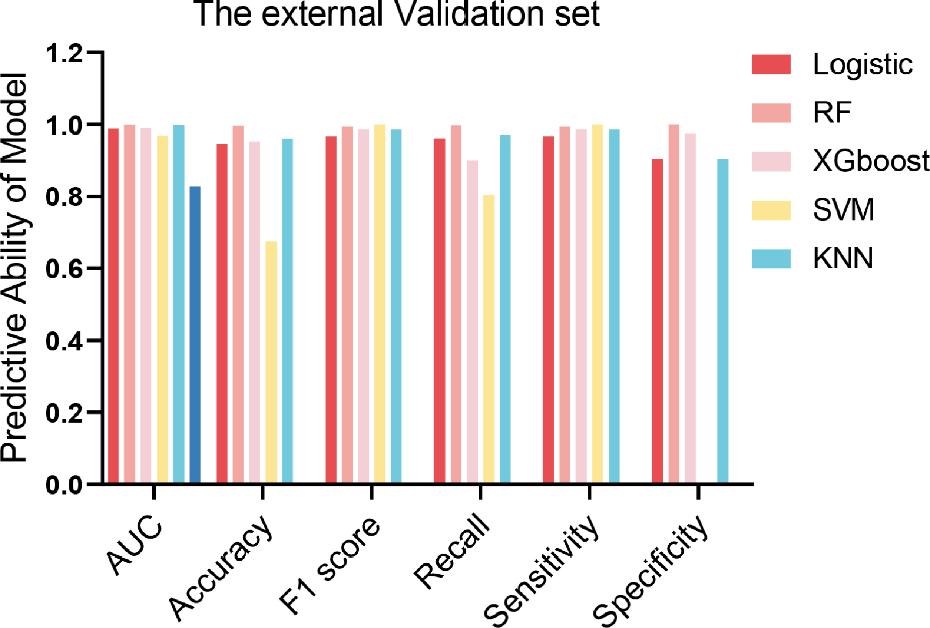


**Figure S1.** Clinical predictive value of 6 machine learning models in the external validation set.


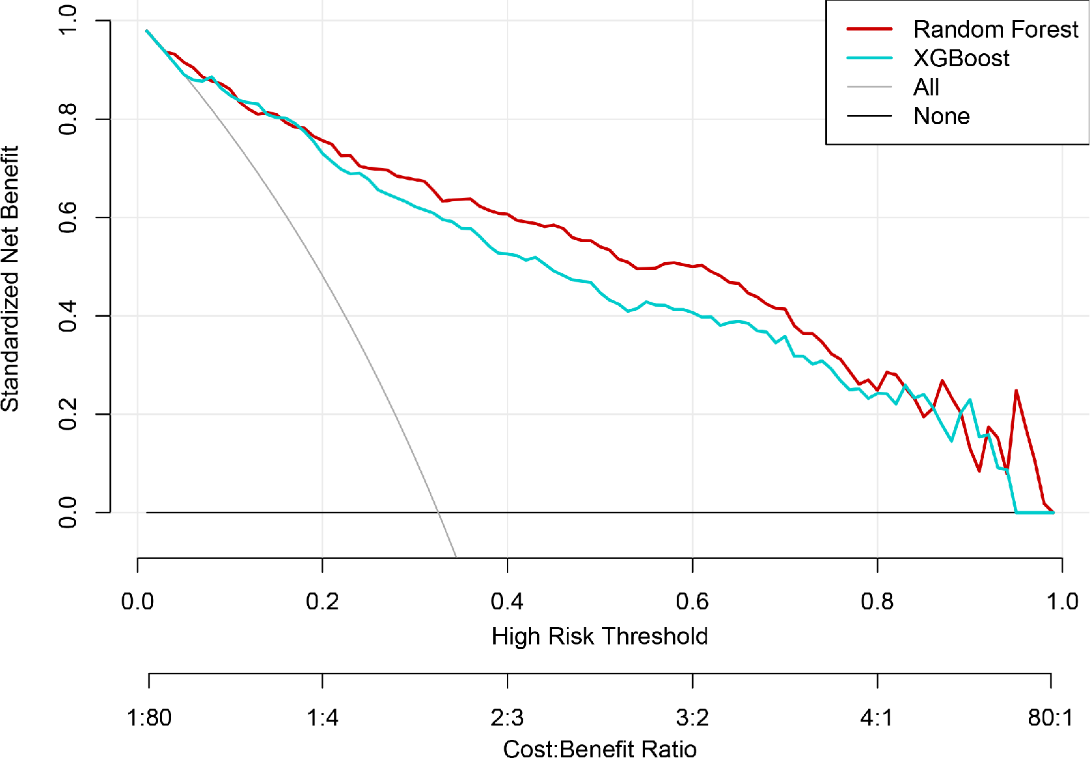


**Figure S2.** Decision curve analysis (DCA) of the APALI prediction models (XGBoost and RF).
